# The burden of hypertension and diabetes mellitus in rural communities in southern Nigeria

**DOI:** 10.11604/pamj.2015.20.103.5619

**Published:** 2015-02-04

**Authors:** Alphonsus Rukevwe Isara, Patrick Otamere Okundia

**Affiliations:** 1 Department of Community Health, University of Benin Teaching Hospital, P. M. B. 1111, Benin City. Nigeria; 2 Department of E.N.T Surgery, Stella Obasanjo Women and Children Hospital, Benin City, Nigeria

**Keywords:** Prevalence, hypertension, diabetes mellitus, rural communities, Nigeria

## Abstract

**Introduction:**

The African region of the world is experiencing a double epidemic of both communicable and non-communicable diseases. The objective of the study was to determine the prevalence of hypertension and diabetes mellitus among adult residents of rural communities in southern Nigeria.

**Methods:**

A community based descriptive cross-sectional study. Adults aged 18 years and above residing in the rural communities who attended a free medical outreach programme were screened for hypertension and diabetes mellitus. Data were collected using a structured interviewer-administered questionnaire.

**Results:**

Of the 845 participants, 349 (41.3%) were aged 50-69 years, 263 (31.1%) were males, and 305 (36.1%) were farmers. Overweight and obesity were found in 184 (21.8%) and 90 (10.6%) of them respectively. The overall prevalence of hypertension was 37.6% (males 43.7%, females 35.1%, p = 0.018) while that of diabetes mellitus was 4.6% (males 1.9%, females 5.8%, p = 0.012). Predictors of hypertension were age ≥ 40 years (OR = 5.04, CI: 2.99 - 8.48), overweight/obesity (OR = 1.56, CI: 1.15 - 2.13) while females are less likely to develop hypertension (OR = 0.72, CI: 0.53 - 0.98). The significant predictor of diabetes mellitus was overweight/obesity (OR = 3.53, CI: 1.78 - 6.98).

**Conclusion:**

The rising prevalence of hypertension and diabetes mellitus is assuming an epidemic level in rural communities in southern Nigeria. There is an urgent need for intensive health education and community surveillance programmes targeted at rural communities in order to achieve prevention and control of these non-communicable diseases in Nigeria.

## Introduction

The African region of the world is experiencing a double epidemic of both communicable and non-communicable diseases. Hypertension and diabetes are among the leading cause of the burden of non-communicable diseases in developing countries. The World Health Statistics 2012 report that one in three adults worldwide, has a raised blood pressure - a condition that causes around half of all deaths from stroke and heart disease while one in 10 adults has diabetes [1]. Hypertension is the commonest co-morbidity of diabetes and verse versa. Both conditions exert a huge financial burden on individuals, families, communities and the health system of any country. In Nigeria, hypertension is the commonest cardiovascular disease reported [2]. Many community based studies have reported varying prevalence rates of hypertension in various parts of the country. A National Non-communicable Disease Survey in 1997 reported an adult hypertension prevalence of 11.4%, with a variation of 14.8% and 9.8% in urban and rural areas respectively [3]. A meta analysis of the prevalence of hypertension from population based studies in south western Nigeria from 1990 to 2009 reported a prevalence ranging from 12.4% to 34.8% with a higher prevalence in men than women and in urban areas than rural areas [4]. However, a similar review of studies on hypertension over the past five decades reported a prevalence ranging from 8% to 46.45%, but with similar prevalence in men and women and higher prevalence in rural than in urban setting [5].

On the other hand, diabetes mellitus (DM) significantly contributes to medical morbidity and mortality worldwide, especially in developing countries like Nigeria. In 2004 and 2010, an estimated 3.4 million people died from the consequences of high fasting blood sugar [6, 7], with more than 80% of these deaths occurring in low- and medium-income countries of the world [8]. In the World Health Organization (WHO) African region in 2011, an estimated 14.7 million adults were suffering from diabetes which resulted in 344,000 deaths and nearly 2.8 billion dollars was spent on the disease by countries in the region [9]. Studies in Nigeria have reported that the prevalence of diabetes varies across different zones of the country but ranges from 2.2 - 9.8% [3, 10–12]. The diabetes statistics of the International Diabetic Federation (IDF) showed that Nigeria has the highest number of people living with diabetes and impaired fasting glucose (IFG) in Africa [13]. We hypothesize that there could be a huge burden of hypertension and diabetes mellitus among adult residents of rural communities in southern Nigeria. Therefore, the objective of this study was to determine the prevalence of hypertension and diabetes mellitus among adult residents of five rural communities in Esan South East Local Government Area of Edo State, in southern Nigeria. Also, we related the socio-demographic characteristics of the study participants and hypertension and diabetes mellitus in order to ascertain the risk factors associated with these conditions.

## Methods

### Study design, setting and population

This community based descriptive cross-sectional study was carried out among adults aged 18 years and above who were residents of five rural communities in Esan South East Local Government Area (LGA) of Edo State, in the southern part of Nigeria. The study was carried out as part of a free medical mission outreach programme held from 16th to 21st December, 2013 at Ewohimi community. Esan South East LGA has a total population of 167,721 comprising 87,535 males and 80,186 females [14]. The LGA is made up of ten political wards but participants for the study were drawn from five wards namely; Ewohimi, Ewatto, Ewossa, Ubiaja and Ohordua. Majority of the inhabitants of Esan South East LGA are farmers and traders. Permission to carry out the outreach programme was sought and obtained from the Chairman of Esan South East LGA and the traditional rulers ("*Enogie*") of the respective communities.

### Data collection

Data was collected using the following instruments:

**Questionnaire:** a structured interviewer-administered questionnaire was used to collect information on the socio-demographic characteristics of the respondents.

### Measurements

**Blood pressure:** blood pressure (BP) was measured using the OMRON Arm-type fully Automatic Digital Blood Pressure Monitor, Model BP - 103H. The BP was measured in the sitting position with an appropriate sized cuff encircling the left arm held at the level of the heart. Hypertension was defined as systolic BP ≥ 140 mmHg and/or diastolic BP ≥ 90 mmHg according to the Joint National Committee on Hypertension (JNC) 7 classification [15].

**Blood sugar:** random blood sugar (RBS) was measured using the ACCU-CHEK Advantage II glucometer, Model (800) 858-8072. Aseptic conditions were maintained throughout the procedure. Diabetes was defined as a random blood glucose > 200 mg/dl (11.1mmol/l) [16].

**Weight and height:** the weight and height of the respondents was measured using a standardized Standiometer. Weight was measured to the nearest 0.5kg with the subject standing motionless on the calibrated scale without footwear. Height was measured with the subject standing in an erect position and head positioned so that the top of the external auditory meatus was level with the inferior margin of the bony orbit. The BMI of the subjects was calculated as weight in kilograms divided by height in meters squared. The BMI was then used to classify the subjects as follows: underweight (BMI < 18.5), normal weight (BMI 18.5 - 24.9), over weight (BMI 25.0 - 29.9) and obese (BMI ≥ 30) according to WHO [17].

### Data analysis

The data collected were checked for completeness, coded and were analyzed using the IBM SPSS version 20 statistical software programme (IBM Corp, Armonk, NY, USA). An initial univariate analysis was conducted for all variables to assess the distribution for each variable to establish whether they are within acceptable range. Categorical variables were summarized using proportions while continuous variables were summarized using means and standard deviations. The primary outcome variables were systolic BP, diastolic BP and blood glucose level while the socio-demographic characteristics and BMI constituted the independent variables. In a bivariate analysis the Fishers exact test was used to test the associations between the demographic variables and BMI of the respondents and their hypertensive and DM status. A multinomial logistic regression analysis was used to identify the predictors of hypertension and DM among the participants.

### Ethical approval

This was an outreach programme in which both preventive and curative health services were provided for members of rural communities. The chairman of the local government area and the head of the respective rural communities gave permission to carry out the outreach programme. Hence no institutional ethical approval was required.

## Results

Data from 845 participants (263 males and 582 females) of the medical mission outreach programme were analyzed. Their mean age (standard deviation (SD) was 56.4 (16.3) years. A higher proportion of the participants 349 (41.3%) were aged 50 - 69 years. Farming (36.1%) and trading (30.7%) were the predominant occupation of the respondents (Table 1). More than half (54.8%) of the participants were of normal weight while 108 (12.8%) were underweight. Overweight and obese constituted 184 (21.8%) and 90 (10.6%) of them respectively. The mean systolic BP of the male participants was 155 (SD = 28) mmHg while that of the females was 153 (SD = 31) mmHg. This difference was not statistically significant (p = 0.417). Also, the mean diastolic BP of the males 89 (SD = 16) mmHg was higher than that of the females 87 (SD = 18) mmHg. Again, this difference was not statistically significant (p = 0.114). However, the mean RBS of the females 123 (SD = 49) mg/dl was significantly higher than the mean RBS of the male participants 114 (SD = 34) mg/dl (p = 0.014). Table 2 describes the prevalence of hypertension and DM in the rural communities. More than a third (n = 319, 37.8%) were hypertensive while only 39 (4.6%) participants were identified to have diabetes mellitus.

**Table 1:** Socio-demographic characteristics of respondents

Variables (n = 845)	Frequency	Percent
**Age group (years**)		
18-29	49	5.8
30-49	224	26.5
50-69	349	41.3
70-90	223	26.4
**Sex**		
Male	263	31.1
Female	582	68.9
**Occupation**		
Farming	305	36.1
Trading	259	30.7
Artisan*	180	21.3
Civil servant	66	7.8
**Others**	35	4.1

* Artisan includes: mechanics, electricians, shoe cobblers, photographers, iron benders, fridge repairers, hairdressers and tailors

**Table 2:** Prevalence of hypertension and diabetes mellitus in the rural communities

Variable (n = 845)	Frequency	Percent
Hypertension	319	37.8
Systolic hypertension	395	46.7
Diastolic hypertension	266	31.5
Diabetes mellitus	39	4.6

Table 3 and Table 4 show that there was a statistically significant association between the demographic characteristics of the participants and their hypertensive and DM status. The proportion of participants who had hypertension increased with increasing age (p = 0.0001) and BMI (p = 0.021). A higher proportion of the male participants were hypertensive when compared to their female counterparts (p = 0.018). As for DM, the proportion of participants who had DM also increased with increasing BMI (p = 0.0001) but DM was more prevalent among participant aged 30 - 49 years followed by those aged 50 - 69 years (p = 0.013). A higher proportion of the female participants than males had DM (p = 0.012). Table 5 shows the results of the multinomial logistic regression for the predictors of hypertension and DM in the rural communities. Participants aged 40 years and above have a five times risk of developing hypertension (OR = 5.04, CI: 2.99 - 8.48) while those who are overweight and obese are more likely to have hypertension (OR = 1.56, CI: 1.15 - 2.13). However, female participants are less likely to develop hypertension (OR = 0.72, CI: 0.53 - 0.98). The only identified significant predictor of DM was the BMI of the participants which showed that those with BMI of 25 and above have about four times likelihood of developing DM (OR = 3.53, CI: 1.78 - 6.98).

**Table 3:** Demographic characteristics and hypertensive status of respondents

Variables	Hypertension		Fishers	P-value
	Yes (%)	No (%)		
**Age group (years)**				
18-29	5 (10.2)	44 (89.8)	39.267	0.0001
30-49	61 (27.2)	163 (72.8)		
50-69	154 (44.1)	195 (55.9)		
70-90	99 (44.4)	124 (55.6)		
**Sex**				
Male	115 (43.7)	148 (56.3)		0.018
Female	204 (35.1)	378 (64.9)		
**Body Mass Index**				
Underweight	30 (27.8)	78 (72.2)	9.680	0.021
Normal	172 (37.1)	291 (62.9)		
Overweight	73 (39.7)	111 (60.3)		
Obese	44 (48.9)	46 (51.1)		

**Table 4:** Demographic characteristics and diabetes mellitus status of respondents

Variables	Diabetes mellitus		Fishers exact	P-value
	Yes (%)	No (%)		
**Age group (years)**				
18-29	0 (0.0)	49 (100)	10.258	0.013
30-49	16 (7.1)	208 (92.9)		
50-69	19 (5.4)	330 (94.6)		
70-90	4 (1.8)	219 (98.2)		
**Sex**				
Male	5 (1.9)	258 (98.1)	-	0.012
Female	34 (5.8)	548 (94.2)		
**Body Mass Index**				
Underweight	0 (0.0)	108 (100)	23.503	0.0001
Normal	14 (3.0)	449 (97.0)		
Overweight	13 (7.1)	171 (92.9)		
Obese	12 (13.3)	78 (86.7)		

**Table 5:** Multinomial logistic regression for the predictors of hypertension and diabetes mellitus in the rural communities

Factors	Hypertension		Diabetes mellitus	
	Odds ratio	95% CI	Odds ratio	95% CI
**Age group (years)**				
< 40*	–	–	–	–
≥ 40	5.04^a^	2.99 – 8.48	2.12^c^	0.74 – 6.14
**Sex**				
Female*	–	–	–	–
Male	0.72^b^	0.53 – 0.98	2.57^c^	0.98 – 6.78
**Body Mass Index**				
< 25*	–	–	–	–
≥ 25	1.56^a^	1.14 – 2.13	3.53^a^	1.78 – 6.98

CI, Confidence interval

* Reference

a P < 0.001

b P < 0.05

c P > 0.05

## Discussion

In this study, we found that the burden of hypertension and DM was high in the rural communities studied, therefore constituting a major public health challenge in these communities. Hypertension increased with increasing age and was more prevalent in male participants and in those who were overweight and obese. On the other hand, the most important significant predictor of DM was a BMI of ≥ 25. The large turnout of people for the medical mission outreach programme in Edo state is a reflection of the weak health care delivery system in Nigeria and many sub-Saharan countries. Thus members of rural communities will catch in on every opportunity to receive free medical care. The mean age of the participants (56.4 years) in this study was far higher than the mean age of 30.7 years reported in a previous study carried out in Edo state, Nigeria [18]. A possible reason for this difference could be the fact that in the previous study respondents were aged 15 years and above while in our study they were aged 18 years and above. The prevalence of underweight in this study was 12.8% while overweight and obesity both constituted 31.8% of the respondents. This showed a double nutritional challenge in these rural communities. Whereas underweight can be explained to have resulted from the high level of poverty and probably the staple food being “pounded yam” which is high in energy content, overweight/obesity could have resulted from the fact that more than two third (68.9%) of our study population were females who are generally more prone to overweight/obesity [19, 20]. This situation underscores the need for nutritional programmes in these communities especially with special emphasis on dietary diversification.

This study revealed a high prevalence of hypertension (37.8%) among residents of these rural communities. This was far higher than the prevalence of 20.2% reported in a previous study by Omuemu et al [18] in a rural community in Edo state. Mezie-Okoye et al [21] reported a prevalence of 18.3% in a rural community in the Niger Delta region of Nigeria, Asekun-Olarinmoye et al [22] reported 13.1% in southwestern Nigeria while Ekwunife et al [23] reported 30.0% in south east, Nigeria. Studies in other countries in sub-Saharan Africa and other parts of the world had also reported lower prevalence of hypertension when compared to our findings [24, 25]. However, our finding was lower than the 42.0%, 44.5% and 46.4% reported by Ulasi et al [26], Ahaneku et al [27] and Onwubere et al [28] respectively all in South Eastern Nigeria. This is a revelation that hypertension is assuming an epidemic dimension in rural communities in Nigeria. There is need for aggressive health education and community surveillance programmes for blood pressure to help cub this rising burden of hypertension especially in rural communities. The high prevalence of isolated systolic hypertension (46.7%) could have resulted from anxiety on the part of the respondents as they have to wait for a long time to be attended to, since in an outreach setting, there are usually a large number of people to be seen daily. The significant predictors of hypertension were age ≥ 40 years, overweight/obesity and male gender. This is consistent with findings from several previous studies [23, 24, 26, 27]. It has been documented in literature that blood pressure increases with increasing age as well as increasing BMI. A probable explanation for the higher prevalence of hypertension among males may be due to modifiable risk factors such as smoking and high alcohol intake which is more common among males compared to females in our environment as documented in a previous study in a rural community in Edo State, Nigeria [29].

The prevalence of DM (defined by random blood sugar > 200 mg/dl) in this study was 4.6%. This is higher than the 2.2% obtained in a national survey in 1997^3^ and the 0.8% reported in rural northern Nigeria [30]. A slightly higher prevalence of 6.0% was found in screening programme in Sokoto, Nigeria [31], however, Mezie-Okoye [32] reported a much higher prevalence of 25.2% in a rural community in south-east Nigeria. Although age, sex and BMI were significantly associated with DM in this study, only overweight/obesity was a significant predictor of diabetes mellitus. This finding of only 10.6% respondents who were obese could possibly explain the prevalence level of DM seen in this study. Also, since majority of the study participants were farmers, trekking long distances to the farm and the farm work itself constituted increased physical activity which is protective against DM. In fact even those who engaged in other occupations such as trading also engage in backyard farming in the rural communities thus sedentary lifestyle may be rare in these rural communities. However, efforts should be made to raise awareness about screening for DM through health education in rural communities to prevent a rise in the prevalence of the condition because most people with DM are not aware that they have the disease.

Our study has some limitations. Only one measurement of BP was used to classify the participants in the study. Also, DM was diagnosed using RBS criteria whereas an FBS would have been a better option. Therefore we were not able to identify those with impaired glucose function. However, this study has raised an alarm on the rising burden of hypertension and DM in rural communities in Edo State, Nigeria. This will serve as a useful tool in the planning of intervention programmes aimed at the control of these non-communicable diseases in rural communities in Nigeria.

## Conclusion

The rising prevalence of hypertension and diabetes is assuming an epidemic level in rural communities in southern Nigeria. The associated factors included increasing age, sex, and overweight/obesity. There is an urgent need for intensive health education and community surveillance programmes targeted at rural communities in order to achieve prevention and control of these non-communicable diseases in Nigeria.
